# Developing a Novel Gene-Delivery Vector System Using the Recombinant Fusion Protein of *Pseudomonas* Exotoxin A and Hyperthermophilic Archaeal Histone HPhA

**DOI:** 10.1371/journal.pone.0142558

**Published:** 2015-11-10

**Authors:** Xin Deng, Guoli Zhang, Ling Zhang, Yan Feng, Zehong Li, GuangMou Wu, Yuhuan Yue, Gensong Li, Yu Cao, Ping Zhu

**Affiliations:** 1 Experimental Center of the Functional Subjects, Basic Medical Scientific Research College, China Medical University, Shenyang, Liaoning, P.R.China; 2 Institute of Veterinary Medicine, The Academy of Military Medical Sciences of PLA, Changchun, Jilin, P.R. China; 3 Key Laboratory for Molecular Enzymology, Jilin University, Changchun, Jilin, P.R.China; 4 Department Biology and Technology of the Agriculture University of Jilin, Changchun, Jilin, P.R.China; 5 Department of Physiology, China Medical University, Shenyang, Liaoning, P.R.China; Fudan University, CHINA

## Abstract

Non-viral gene delivery system with many advantages has a great potential for the future of gene therapy. One inherent obstacle of such approach is the uptake by endocytosis into vesicular compartments. Receptor-mediated gene delivery method holds promise to overcome this obstacle. In this study, we developed a receptor-mediated gene delivery system based on a combination of the *Pseudomonas* exotoxin A (PE), which has a receptor binding and membrane translocation domain, and the hyperthermophilic archaeal histone (HPhA), which has the DNA binding ability. First, we constructed and expressed the rPE-HPhA fusion protein. We then examined the cytotoxicity and the DNA binding ability of rPE-HPhA. We further assessed the efficiency of transfection of the pEGF-C1 plasmid DNA to CHO cells by the rPE-HPhA system, in comparison to the cationic liposome method. The results showed that the transfection efficiency of rPE-HPhA was higher than that of cationic liposomes. In addition, the rPE-HPhA gene delivery system is non-specific to DNA sequence, topology or targeted cell type. Thus, the rPE-HPhA system can be used for delivering genes of interest into mammalian cells and has great potential to be applied for gene therapy.

## Introduction

Gene therapy refers to transgenosis, which transfers a gene encoding a function protein into a cell and expresses it successfully in the target cell. The efficient transfers of DNA into cells often use a viral vector via a receptor-mediated endocytosis and/or liposome-mediated transfection *in vitro* [1–6]. However, these viral vector-based gene delivery systems have some unavoidable disadvantages [7]. Although nonviral gene delivery systems are much less immunogenic and cytotoxic than those of viral vector systems, they bear low gene transfer efficiency [8].

Receptor-mediated gene delivery can offer high efficiency in gene transfer, but several technical problems still need to be overcome. One of the key problems is that the foreign components have been compartmentalized and subsequently degraded by the lysosomes after internalizing them into cells [9,10].

To develop gene delivery system, *Pseudomonas* exotoxin A (PE) is employed as a part of the novel gene delivery vehicle, rPE-HPhA. PE is a single chain protein and consists of three domains. Domain Ia (residues 1–252) is responsible for cell recognition. Domain II (residues 253–364) is involved in translocation of the toxin across membranes. Domain Ib (residues 365–404) is still unclear for its function. Domain III (residues 405–613) may catalyze the ADP-ribosylation of elongation factor-2, which arrests protein synthesis and results in cell death [11–16]. When PE goes through the cell membrane into the cytosol, the deletion of domain Ib and domain III doesn’t have an influence on its recognition and translocation [17,18]. The domain Ia (receptor binding domain) of PE is a highly specific ligand to the LDL/α2-macroglobulin receptor, which exists in all mammalian cells [19–21]. The membrane translocation domain of PE may increase the translocation efficiency of DNA. Thus, domain Ia and domain II of PE may be used as a general DNA delivery vehicle that has no cell-type specificity.

*Pyrococcus horikoshii* strain OT3 is a hyperthermophilic archaeon [22]. Two open reading frames of its genome, PHS051 (HPhA) and PHS046 (HPhB), were putative archaeal histones [23]. Their gene sequences show that the HPhA gene may encode a histone-like protein. The result of gel retardation assay demonstrated that purified recombinant HPhA forms a large carrier-complex of DNA aggregates and, therefore, may increase the transfection efficiency [24]. The binding affinity of HPhA to DNA didn’t limit to DNA sequences or topological forms [25,26]. Thus, we use HPhA gene as a DNA binding protein domain to construct a gene delivery vehicle by fusing with domain Ia and domian II of PE.

In this study, we fused both receptor binding and membrane translocation domains of PE with the DNA binding region of HPhA. We demonstrated that the novel DNA delivery system not only possesses target specificity but also delivers foreign DNA with no specificity of cell-type. Thus, this DNA delivery protein may be used in target gene delivery systems and biochemical studies.

## Materials and Methods

### Reagents

Plasmid pET26b was purchased from Novagen. BH5α was purchased from TaKaRa. *E*. *coli* BL21(DE3)-codon plus and Lipofectamine 2000 were purchased from Invitrogen, NY. pET11a-HPhA was a gift from Prof. Yan Feng (Jilin University). pET26b-PE was provided by Prof. Guoli Zhang (Changchun, China). Anti-HPhA antiserum was provided by Dr. Ling Zhang (Changchun, China). Microbiological culture media were purchased from Oxoid, Hampshire, UK. Cell culture media, RPMI 1640, heat-inactivated fetal calf serum and trypsin-EDTA were supplied by Gibco BRL, NY. Other chemicals unless otherwise specified were purchased from Sigma-Aldrich, Sydney, Australia.

### Construction of DNA carrier vehicle

To construct the plasmid encoding the multifunctional fusion protein containing the domain Ia and domain II of PE, and the DNA binding domain of recombinant HPhA, the DNA fragment encoding amino acids 1–364 of PE from *Pseudomonas aeruginosa* was amplified with polymerase chain reaction (PCR) using plasmid pET26a-PE as the template, which included whole PE gene sequence. Two oligonucleotide primer sequences used are: 5′-GGAATTCCATATGGCCGAGGAAGCCTTC-3′ and 5′-CGGAATTCCGCGCCGGCCTCGTC-3′ with *Nde*I and *Eco*RI restriction sites at the 5′ and 3′ ends of the PE fragment. The PCR products with the correct size were purified and digested with the corresponding restriction enzymes, and cloned into pET26b, resulting in pET26b-PE_Ia+II_.

The DNA fragment encoding the DNA-binding domain of HPhA was PCR amplified using plasmid pET11a-HPhA as the template [24]. Two oligonucleotide primer sequences are 5′-CGGAATTCGTGTGGATGATGGGAGAA-3′ and 5′-CCCAAGCTTTCAGCTCTTAATAGCGAGC-3′ with *Eco*RI and *Hin*dIII restriction sites at the 5′ and 3′ ends. The amplification products of correct size were purified, digested and cloned into pET26b-PE_Ia+II_, resulting in pET26b-PE_Ia+II_-HPhA, which encoding the recombinant protein rPE-HPhA that comprises of the receptor binding and membrane translocation domains of PE, and the DNA-binding domain (70 amino acid residues) of HPhA.

### Expression and purification of fusion proteins

Fusion protein rPE-HPhA were expressed in *E*. *coli* BL21 (DE3)-codon plus and were grown at 37°C in LB medium containing 50 μg/ml of kanamycin to OD600 = 0.6~0.8. Protein expression was induced by addition of 1.0 mmol/L isopropyl-b-D-thiogalactopyranoside (IPTG) at 34°C for 300 min. Cells were harvested by centrifugation, the pellet was resuspended in 30 mmol/L Tris-HCl, pH 8.0.

The cells were lysed by ultrasonication. Cell lysates were centrifugated at 12,000 rpm for 10 min at 4°C, and the supernatant was precipitated by 35% ammonium sulfate, and then digested by DNase I, centrifugated at 10,000 rpm for 20 min at 4°C. The sediment was resuspended in 30 mmol/L Tris-HCl, pH 8.0 containing 8 mol/L urea for 2 h at 4°C. The samples were loaded into Blue chelating HP column under the condition of 30 mmol/L Tris-HCl, 4 mol/L urea pH 8.0. Unbound proteins were removed by washing the column with equilibration buffer. But bound proteins were eluted by increasing the sodium chloride concentration of the buffer to 360–600 mmol/L. The eluted fractions were dialyzed against to 30 mmol/L Tris-HCl, 4 mol/L urea pH 8.0. The dialyzed proteins were loaded into Heparin chelating HP column under the condition 30 mmol/L Tris-HCl, 4 mol/L urea pH 8.0, and then eluted against to 30 mmol/L Tris-HCl pH 8.0 for 18 h, in order to redenature the aim proteins. Unbound proteins were removed by washing the column with equilibration buffer. But bound proteins were eluted by increasing the sodium chloride concentration of the buffer to 280–500 mmol/L. The eluted fractions were dialyzed against to 30 mmol/L Tris-HCl pH 8.0. Eluted products were examined by 12% SDS-PAGE.

### Immunoblotting assays

Immunoblottined was carried out as described previously [20,27]. In brief, HeLa cells were treated with rPE-HPhA fusion proteins (concentration: 434.8 nmol/L) in 6-well tissue culture plates with a density of 1×10^4^ cells/well and incubated overnight at 37°C, 5% CO2. The culture medium was exchanged to fresh medium of 2 ml/well for another 12 h. HeLa cells without rPE-HPhA pretreated were used as control group. Experimental groups were purified rPE-HPhA fusion proteins and HeLa cells pretreated with rPE-HPhA. Purified rPE-HPhA and Cell lysates were fractionated by sodium dodecyl sulfate polyacrylamide gel electrophoresis (SDS-PAGE) and transferred onto nitrocellulose membranes (PALL Corporation) [28–30]. The membrane was incubated with the mouse anti-HPhA mAb (1:1000 dilution) overnight at 4°C, followed by reaction with goat anti-mouse IgG horseradish peroxidase conjugated secondary antibody for 1 h at room temperature. After washing, bound secondary antibody was visualized using a chemiluminescent detection regent (Thermo).

### Cytotoxicity of fusion proteins

HeLa cells with PE receptor-binding activity were seeded in 96-well tissue culture plates at a density of 1×10^4^ cells per well and incubated overnight at 37°C, 5% CO_2_ in fresh RPMI medium with 10% fetal calf serum. Fusion proteins rPE-HPhA (concentrations: 33 nmol/L, 65 nmol/L, 129 nmol/L, 260 nmol/L, 510 nmol/L, 1023 nmol/L and 2045 nmol/L) were added to each well, respectively. The experiments were repeated for a minimum of three times. The results were analyzed after 24 h of incubation.

### Receptor competition activity of PE and rPE-HPhA

HeLa cells were seeded in 96-well tissue culture plates at 1×10^4^ cells/well, and incubated overnight at 37°C, 5% CO_2_. PE, as the concentration of IC_50_ (3.75 nmol/L/well), was mixed with different molar concentrations of rPE-HPhA proteins and incubated for 5 min. The ratios of rPE-HPhA and PE proteins were 0, 9, 17, 34, 69, 136, 273, and 545, respectively. These mixtures were then added into 96-well plates. All experiments were repeated for a minimum of three times. The results were analyzed after 24 h of incubation.

### Immunohistochemical analysis of fusion protein rPE-HPhA transfections

To detect if HPhA was delivered into cells, HeLa cells were treated with rPE-HPhA fusion proteins (concentration: 434.8 nmol/L) in 6-well tissue culture plates with a density of 1×10^4^ cells/well and incubated overnight at 37°C, 5% CO2. The culture medium was replaced with fresh medium of 2 ml/well for another 12 h. HeLa cells without rPE-HPhA pretreated were used as control [31]. HeLa cells were then washed 5 times with PBS and fixed. Fixed cells were incubated with the anti-HPhA mAb (1:1000 dilution, mouse) overnight at 4°C, followed by incubation with goat anti-mouse IgG horseradish peroxidase conjugated antibody as secondary antibody and stained with diaminobenzidine (DAB). Images were taken under light microscope.

### Immunofluorescence analysis of fusion protein rPE-HPhA carrying DNA

CHO cells with PE receptor-binding activity were seeded in 6-well tissue culture plates at a density of 1×10^4^ cells/well and grown overnight at 37°C. The growth medium was changed to fresh medium with 2 ml/well at 4 h before addition of protein-DNA complexes. Complexes were prepared by pre-incubation of 2 μg pEGFP-C1 plasmid encoding the green fluorescence protein (GFP) together with 4μg fusion protein in 30 mmol/L Tris-HCl pH 7.5 for 30 min at room temperature. The complexes were added into 1.4 ml RPMI-1640 medium to form the protein-DNA complexes for transfection. And then CHO cells were incubated with the protein-DNA complexes overnight at 37°C, 5% CO_2_ for 12 h. The supernatant was removed. CHO cells were futher incubated successively with 2 ml RPMI-1640 medium for another 40 h. The expression of GFP was observed with fluorescence microscope. Cationic liposomes, plasmids and blank treatments were used as control groups.

Transfection of cationic liposomes was used as a positive control. 3μl Lipofectamine 2000 was mixed with 2 μg pEGFP-C1 in 100 μl RPMI-1640 medium for 30 min at room temperature. Cells were cultured with the transfected complexes in 1.4 ml RPMI-1640 medium without serum at 37°C, 5% CO_2_ for 12 h. And then 2 ml RPMI-1640 medium with 10% fetal calf serum replaced the medium to incubate the cells for next 40 h. 2 μg pEGFP were added for incubated the same time as another negative control. The images were observed under fluorescence microscope. Efficiency of transfection was calculated based on the numbers of fluorescent cells per 500 cells.

### Statistical analysis

Data are presented as mean ± SEM of separate experiments (n > 3) and compared by one-way analysis of variance with SPSS 13.0 software (SPSS, Chicago, IL, USA). *p* < 0.05 was considered statistically significant.

## Results

### Construction and purification of the DNA carrier vehicle

**Fig 1:**
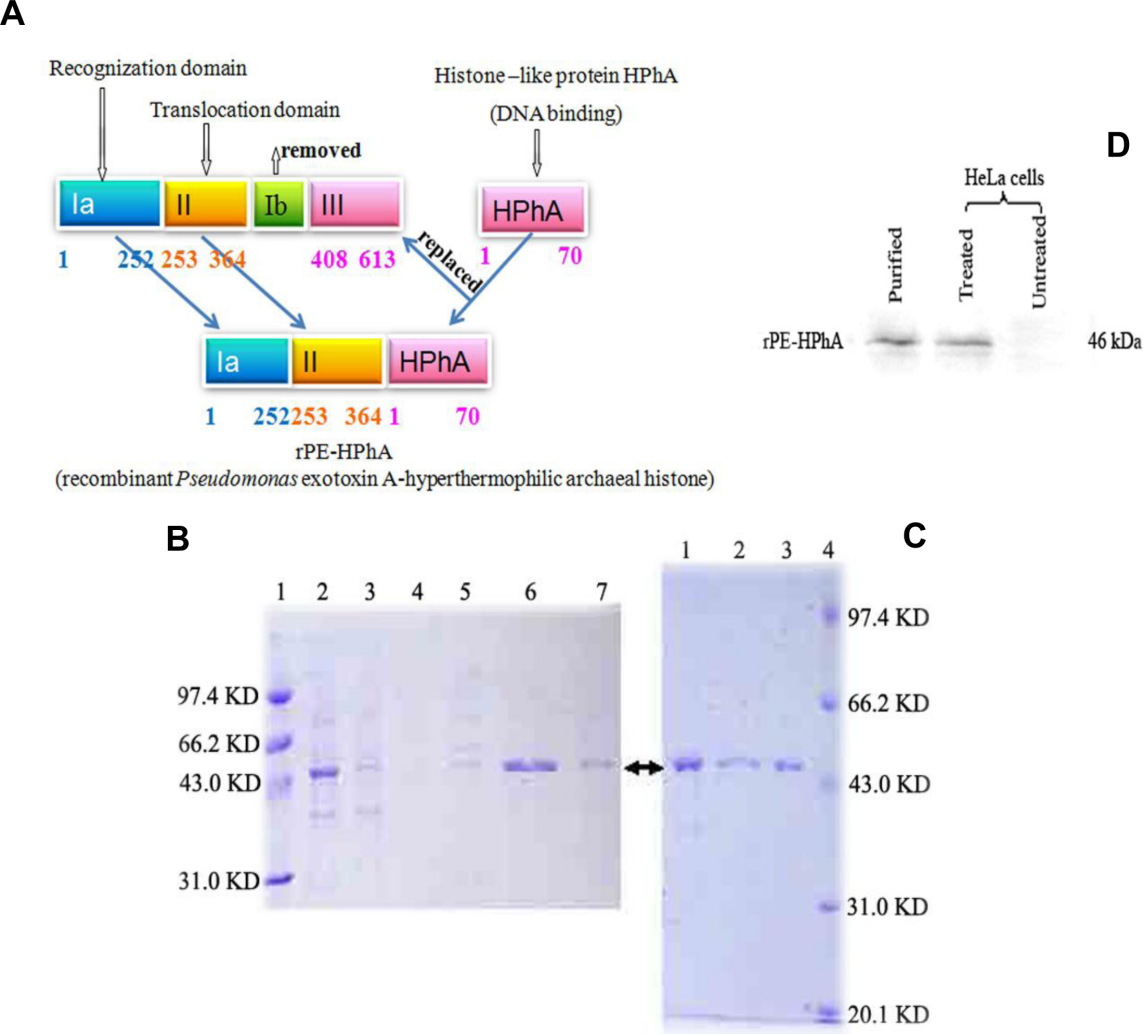
Construction and expression of rPE-HPhA fusion protein. A) Schematic representation of fusion proteins, which consists of an N-terminal amino acids 1–364 of PE and 70 amino acids of HPhA. B) SDS-PAGE of rPE-HPhA protein purified with Blue Chelating Sepharose Fast Flow. Lane 1: Marker; Lane 2: Sample digested with DNase I; Lane 3: Unbound proteins eluated with equilibration buffer; Lane 4, 5, 6 and 7: Bound protein eluated with sodium chloride (from 360 to 600 mmol/L). C) SDS-PAGE of rPE-HPhA protein which is further purified with Heparin Chelating Sepharose FF. Lane 1: The proteins eluated with 0.1 mol/L NaOH; Lane 2: Denatured fusion proteins; Lane 3: Desalted samples; Lane 4: Marker. D) Immunoblot analyses of rPE-HPhA.

The plasmid for overexpression and purification of the fusion protein rPE-HPhA, which comprises of the receptor binding domain and membrane translocation domain of PEA and the DNA binding region of HPhA, was constructed as described in Materials and Mathods (Fig 1A). The fusion protein induced by IPTG was expressed by *E*. *coli* BL21(DE3)-codon plus. The purified fusion proteins were observed to show approximate homogeneity (Fig 1B). Recombinant fusion proteins were purified by Heparin column (Fig 1C), and the purified fusion protein was confirmed with immunoblotting results with anti-HPhA monoclonal antibody (Fig 1D).

### Cytotoxicity of fusion proteins

**Fig 2:**
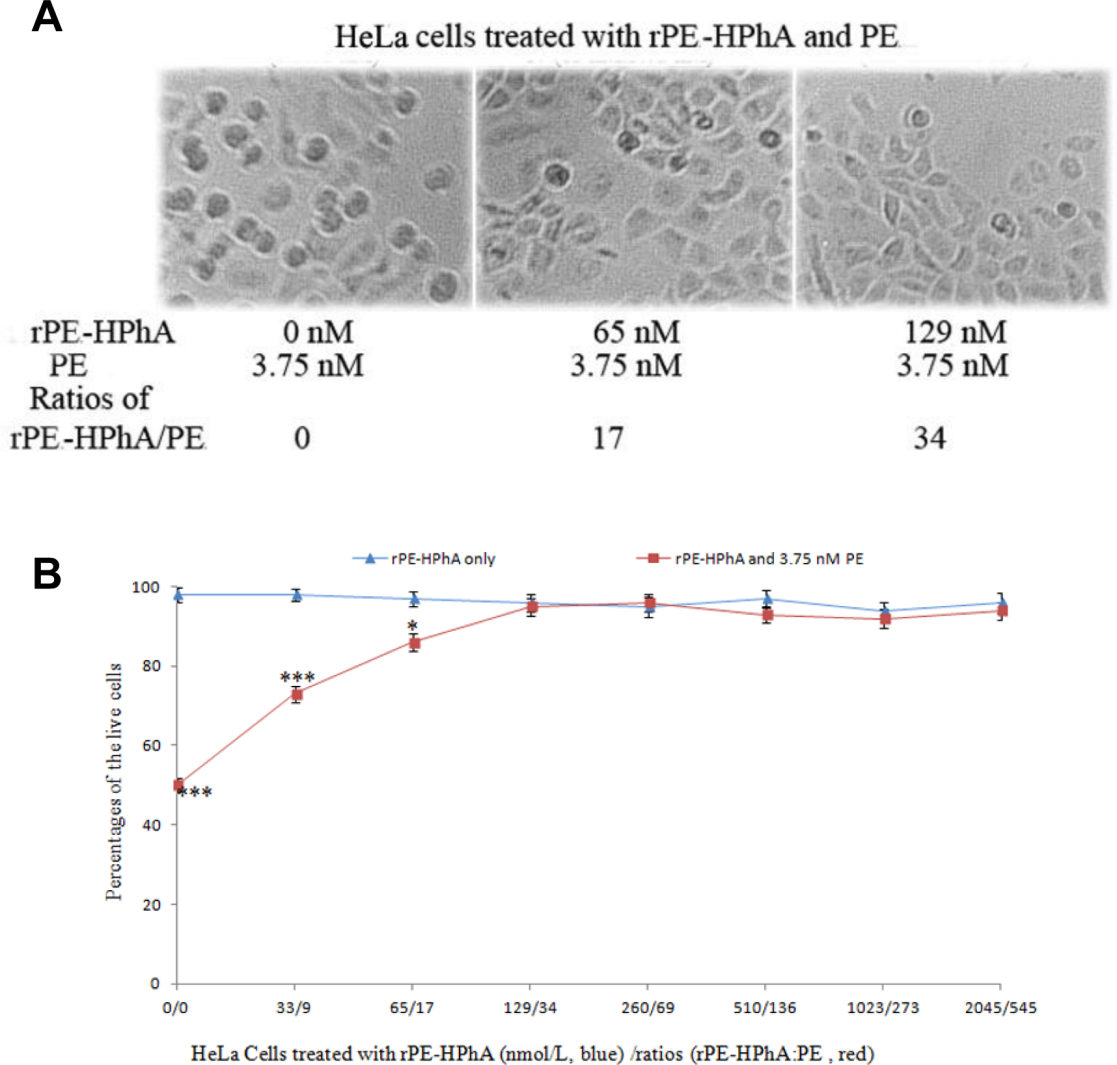
Cytotoxicity of rPE-HPhA. A) Effect of rPE-HPhA (0, 65 and/or 129 nmol/L) and PE (3.75 nmol/L) on HeLa cells killing. All images were magnified 100 times. B) Percentages of the live HeLa cells in different concentrations of rPE-HPhA analyzed by cell count. Blue bars represented the percentages of the living cells under the treatment of rPE-HPhA. Red bars represented the percentages of the living cells under the combined treatment of rPE-HPhA and PE. Asterisk represents the level of significance between low concentrations of rPE-HPhA (0, 33 and/or 65 nM) versus high concentrations of rPE-HPhA (more than 129 nM) in the presence of PE (3.75 nM) (***p < 0.001; *p < 0.05). Error bars represent mean ± SEM of three independent experiments.

The cytotoxicity of the rPE-HPhA fusion protein was investigated by incubating HeLa cells with various concentrations of the recombinant proteins from 33 nmol/L, 65 nmol/L, 129 nmol/L, 260 nmol/L, 510 nmol/L, 1023 nmol/L to 2045 nmol/L, respectively. The results showed that the recombinant was no toxicity effects on treated HeLa cells (Fig 2, blue columns).

### Receptor competition activity of rPE-HPhA and PE

The receptor-binding ability of the fusion protein was confirmed with a competition experiment by living cell counts under light microscope. Our results showed that increasing concentrations of rPE-HPhA did not induce death of HeLa cells (Fig 2B, blue). Also, increasing concentrations of the rPE-HPhA (from 33 nM to 2045 nM) in the presence of PE (3.75 nM) led to increased percentages of cell survival (Fig 2). When the ratio of rPE-HPhA to PE reached about 17, the percentage of the live cells increased significantly. When the ratio reached about 34, the percentage of the live cells almost reached its maximal value. Our results indicated that rPE-HPhA could completely restrain the cytotoxicity of PE, when the ratio of concentration was more than 34 (Fig 2B, red columns).

### rPE-HPhA mediated gene transfer requires the activity of the PE translocation domain

**Fig 3:**
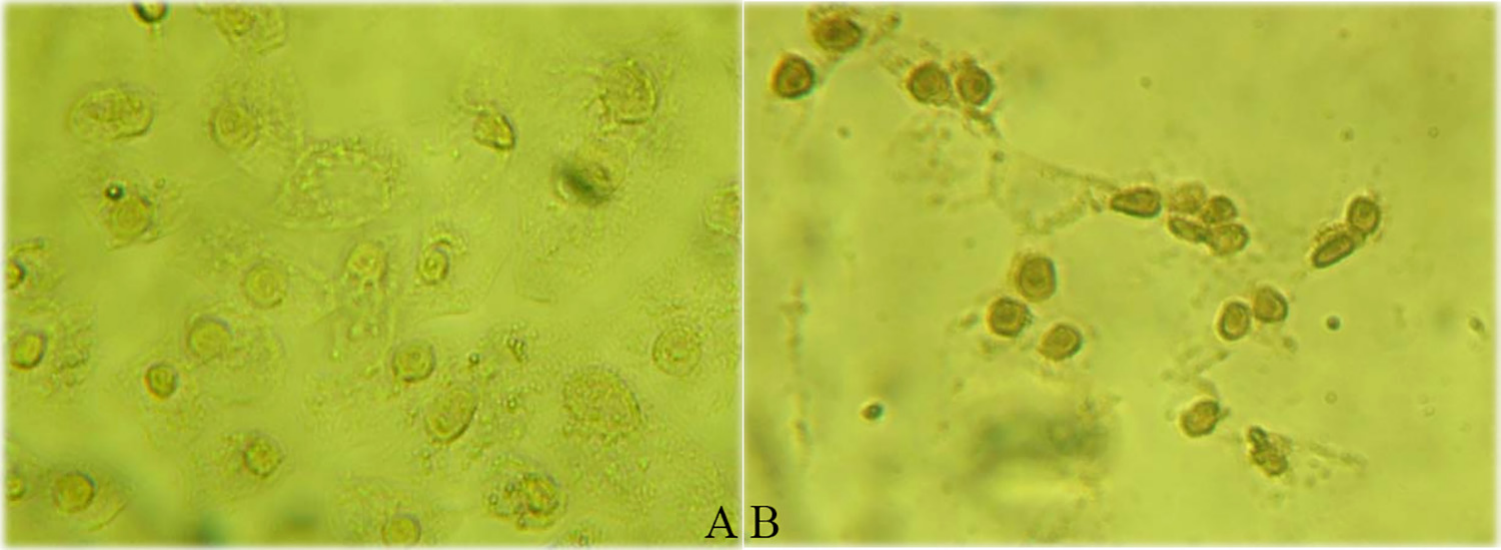
Transferring HPhA from the fusion protein rPE-HPhA into cytosol with immunohistochemical method. The fixated cells were incubated with anti-HPhA mAb followed by interaction with goat anti-mouse IgG horseradish peroxidase conjugated antibody as secondary antibody, and stained with diaminobenzidine (DAB). A) Untreated HeLa cells without immunohistochemical staining observed. B) Immunohistochemical staining was observed in HeLa cells treated with fusion protein rPE-HPhA.

In the rPE-HPhA fusion protein, the translocation domain derived from the PE was included for endosome escape. The N-terminal recognization domain derived from the Ia of PE targets mammalian cells. The C-terminal enzymatic domain of the toxin PE was replaced by the DNA-binding of HPhA. To confirm that the PE recognization domain and translocation domain in the fusion protein were functionally active, fusion proteins were added to HeLa cells and were examined by immunohistochemical method. Our data showed that rPE-HPhA was observed in treated cells but not in untreated control (Fig 3).

### Detection of rPE-HPhA-mediated gene transfer in CHO cells

**Fig 4:**
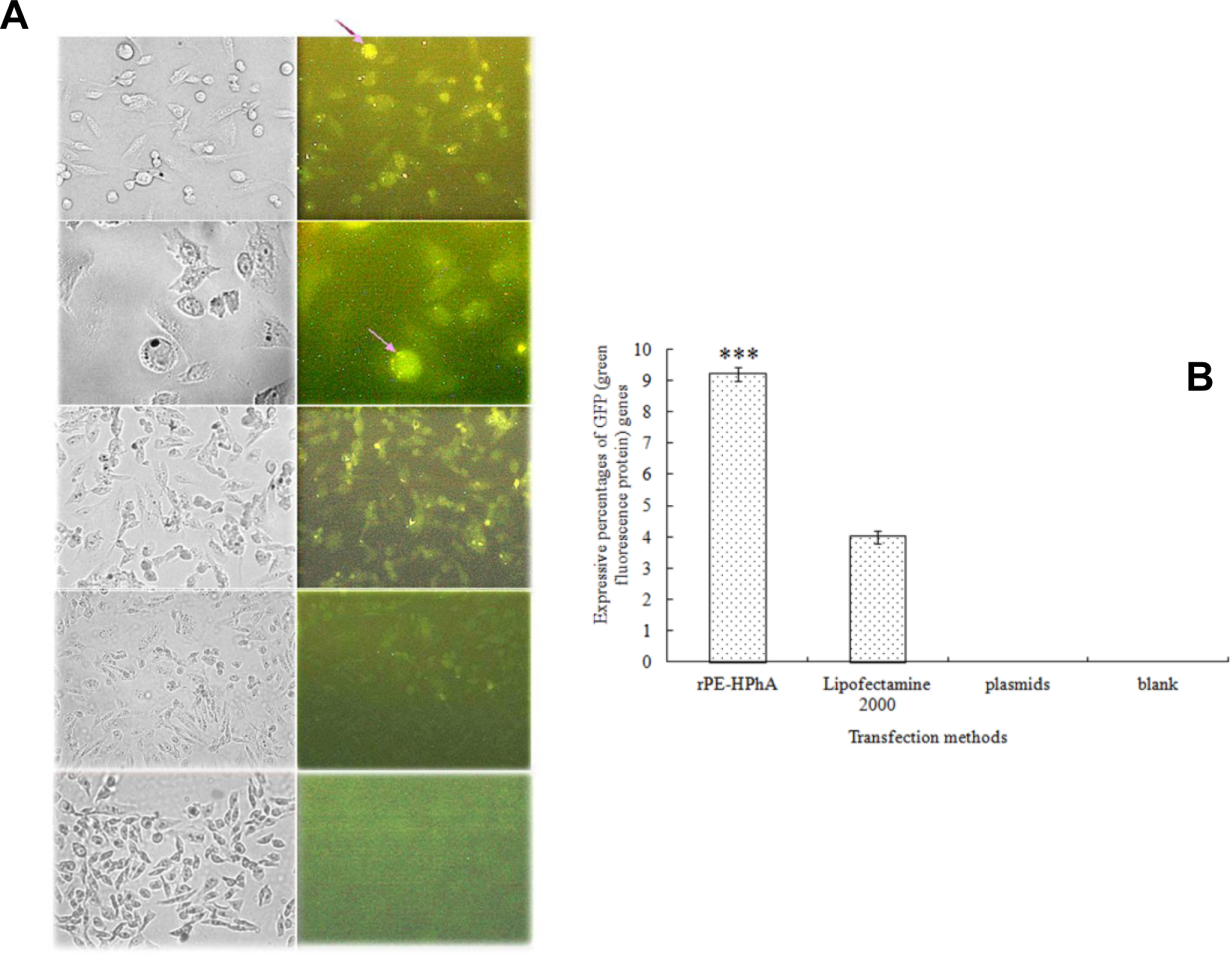
The transfection ability of the DNA delivery protein rPE-HPhA in CHO cells. A) The first and second panels: CHO cells were treated with a mixture of plasmid pEGFP-C1 (2ug) and rPE-HPhA (4ug). EGFP expression was detected with fluorescent microscope. Arrows indicate the green fluorescence spot in transfected cell; The first panel: visible light 200 × (left) and fluorescence 200 × (right); The second panel: visible light 400 × (left) and fluorescence 400 × (right); The third panel: CHO cells was treated with a mixture of EGFP (2ug) and lipofectamine 2000 (3ul). Visible light 200 × (left) and fluorescence 200 × (right); The fourth panel: CHO cells were treated with plasmid EGFP alone. Visible light 200 × (left) and fluorescence 200 × (right); The fifth panel: Untreated cells, no green fluorescence was detected in the 4^th^ and 5^th^ panels. B) Transfer efficiency of rPE-HPhA, Lipofectamine 2000, pEGFP-C1 and/or Untreated cells. Asterisk represents the level of significance between rPE-HPhA groups versus Lipofectamine 2000 groups (***p < 0.001). Error bars represent mean ± SEM of three independent experiments.

The fusion protein-mediated gene transfer was analyzed using pEGFP-C1 which encodes the gene of green fluorescent protein. Purified fusion protein and pEGFP-C1 DNA were co-incubated to form protein-DNA complexes. As a control, CHO cells were also treated with a mixture of pEGFP-C1 and Lipofectmine 2000 or plasmid pEGFP-C1 alone. The cells were incubated with transfection complexes overnight, then were changed to a fresh medium. Cells were incubated for another 48 h, harvested, and analyzed under fluorescent microscope (Fig 4A).

DNA transfer efficiency of rPE-HPhA was further quantified by counting the cells that emitted green fluorescence. The transfer efficiency of rPE-HPhA was on an average of 46 cells per 500 cells, which was much higher than that of transfection mediated by liposome (19 cells per 500 cells); The plasmid pEGFP-C1 only was hardly delivered into cells. These results showed that rPE-HPhA was capable of delivering DNA. To compare the efficiency of rPE-HPhA-mediated gene transfer with that mediated by cationic lipids Lipofectamine 2000, we found that rPE-HPhA could induce higher levels of green fluorescent protein and have higher transfection efficiency than that of the liposome. In particular, rPE-HPhA had little toxicity to the tested cells (Fig 4B).

## Discussion

Development of medical treatments has been increasingly focused on the etiology of a disease rather than on the treatment of symptoms. Gene vector, as the tool of gene therapy, is the determinant for the outcome of gene therapy. Many gene delivery vectors have also been reported [2,3]. These vectors deliver genes of interest passively, or bind to DNA (in the case of cationic polymer and polylysine) and are taken up by endocytosis. These systems have inherent limitations and disadvantages, regardless either viral or non-viral vectors [32,33]. Although gene transfers via receptors or mediated by DEAE-dextran may enhance the function of endocytosis, any successful application has not yet been reported [34,35]. A novel gene delivery method still needs to be developed.

Receptor-mediated DNA transfer method relies on the binding of DNA-containing ligand to a receptor, and the subsequent receptor-mediated endocytosis [36–39]. Thus, a highly specific and efficient ligand should increase the efficiency of DNA transfer. Since all mammalian cells bear the receptor of PE, a DNA delivery vehicle containing Ia domain of PE may have little cell-type specificity.

In the present study, we fused Ia and II domain of PE with the HPhA gene for improving the efficiency of DNA delivery by Ia receptor-mediated DNA transportation. We detected that the rPE-HPhA/pEGFP-C1 complexes were successfully transfected into CHO cells. Moreover, the efficiency of transfection was higher than that of lipofectamine-mediated method. Since rPE-HPhA has little cytotoxicity, the purified fusion protein is suitable for delivering DNA into cells.

The translocation domain of PE toxin into cytosol has been shown to increase the transfer efficiency of DNA [40]. By integrating the receptor binding and membrane translocation domains of PE, the modified PE can bind to receptor and transfer to cells. Since the ADP-ribosylation domain of PE has been replaced by HPhA gene, the rPE-HPhA can then bind DNA without cell killings. The advantage of recombinant rPE-HPhA binding to DNA is that it has no sequence or topological specificity towards DNA. The complex substance of fusion protein rPE-HPhA and DNA should bind to PE receptors on the target cells and then enter cells via receptor-mediated endocytosis. With immunohistochemical analysis, we detected that HPhA was transferred into HeLa cells by this general DNA deliver system. We observed that the transfer efficiency of plasmid DNA pEGFP-C1 into CHO cells was higher than that of liposome transfection. So rPE-HPhA is a very promising gene delivery vector to mediate gene transfer *in vitro*.

Taken together, our results suggest that the rPE-HPhA vector system has several advantages including high efficiency, nontoxicity, none cell-type targeting, and bypassing the endocytic pathway to minimize DNA degradation. This system possesses a great potential to be used in gene therapy or cancer therapy. More explorations need to be conducted in the future in order to optimize its targeting function in gene therapy, for example, to construct a LHRH-HPhA fusion protein for targeting some tumor cells [41–45].
